# Genetic Analysis of H5N1 High-Pathogenicity Avian Influenza Virus following a Mass Mortality Event in Wild Geese on the Solway Firth

**DOI:** 10.3390/pathogens13010083

**Published:** 2024-01-17

**Authors:** Craig S. Ross, Alexander M. P. Byrne, Sahar Mahmood, Saumya Thomas, Scott Reid, Lorna Freath, Larry R. Griffin, Marco Falchieri, Paul Holmes, Nick Goldsmith, Jessica M. Shaw, Alastair MacGugan, James Aegerter, Rowena Hansen, Ian H. Brown, Ashley C. Banyard

**Affiliations:** 1 Virology Department, Animal and Plant Health Agency (APHA), Addlestone KT15 3NB, UK; 2 Animal Health and Welfare Advice, Animal and Plant Health Agency (APHA), Addlestone KT15 3NB, UK; 3 ECO-LG Ltd., Crooks House, Mabie, Dumfries DG2 8EY, UK; 4 APHA Diseases of Wildlife Scheme, Shrewsbury Veterinary Investigation Centre, Shrewsbury SY1 4HD, UK; 5 NatureScot, Great Glen House, Inverness IV3 8NW, UK; 6 National Wildlife Management Centre, Animal and Plant Health Agency (APHA), York YO41 1LZ, UK; 7 WOAH/FAO International Reference Laboratory for Avian Influenza, Swine Influenza and Newcastle Disease, Animal and Plant Health Agency (APHA), Addlestone KT15 3NB, UK

**Keywords:** high-pathogenicity avian influenza, H5N1, mass die-off, genetic stability

## Abstract

The United Kingdom (UK) and Europe have seen successive outbreaks of H5N1 clade 2.3.4.4b high-pathogenicity avian influenza virus (HPAIV) since 2020 peaking in the autumn/winter periods. During the 2021/22 season, a mass die-off event of Svalbard Barnacle Geese (*Branta leucopsis*) was observed on the Solway Firth, a body of water on the west coast border between England and Scotland. This area is used annually by Barnacle Geese to over-winter, before returning to Svalbard to breed. Following initial identification of HPAIV in a Barnacle Goose on 8 November 2021, up to 32% of the total Barnacle Goose population may have succumbed to disease by the end of March 2022, along with other wild bird species in the area. Potential adaptation of the HPAIV to the Barnacle Goose population within this event was evaluated. Whole-genome sequencing of thirty-three HPAIV isolates from wild bird species demonstrated that there had been two distinct incursions of the virus, but the two viruses had remained genetically stable within the population, whilst viruses from infected wild birds were closely related to those from poultry cases occurring in the same region. Analysis of sera from the following year demonstrated that a high percentage (76%) of returning birds had developed antibodies to H5 AIV. This study demonstrates genetic stability of this strain of HPAIV in wild Anseriformes, and that, at the population scale, whilst there is a significant impact on survival, a high proportion of birds recover following infection.

## 1. Introduction

High-pathogenicity avian influenza virus (HPAIV) subtype H5Nx, clade 2.3.4.4b, caused extensive epizootics during the autumn/winter period of 2020/2021, 2021/2022 and continued through 2022/2023 in the United Kingdom (UK), Europe, North America and is now considered to be trans-global with extensive impacts on wild bird populations [1,2,3,4]. Whilst the emergence of cases in successive years was unusual within the UK, the seasonal pattern of disease followed that seen previously and was associated with the colder months and the arrival or presence of over-wintering migratory wildfowl in the UK and northern Europe (September–March inclusive). High pathogenicity H5N8 virus subtypes predominated in wild birds during the 2020/2021 winter epizootic, although other H5Nx subtypes were occasionally detected in both captive and wild birds [5]. Exceptionally, in the summer (July and August 2021), between successive winter epizootics, HPAIV H5N1 infection caused multiple, independent mass mortality events in great skuas (*Stercorarius skua*) on islands off the northern coast of Scotland [2]. This detection of HPAIV H5N1 over summer was part of the largest avian influenza virus (AIV) epizootic observed to date across Europe and the UK, resulting in substantial numbers of outbreaks in captive birds and a dramatic increase in confirmed cases in wild birds (>1700 detections in Great Britain (GB), October 2021–September 2022 [4]). Currently in GB, two passive surveillance schemes are utilized to track AIV in wild birds. One is an ad hoc system reliant on members of the public observing and subsequently reporting dead wild birds to the Department for Environment, Food & Rural Affairs (Defra). The second engages wild bird conservation sites where workers periodically survey sites and recover dead wild birds. In both cases, wild birds are submitted to the Animal and Plant Health Agency (APHA), although a triage scheme limits the number tested to avoid unnecessary repeat sampling, using species, location and date as factors. Consequently, only a small subset of birds is routinely tested, with testing predominantly concerned with key indicator groups (e.g., waterfowl and raptors) and is opportunistic in its nature. This will therefore mask the true epidemiological consequences of HPAIV in wild birds.

During the 2021/22 and 2022/23 epizootic in the UK, there have been three dominant genotypes of H5N1 HPAIV observed across wild birds and poultry: genotype C (subsequently described as AIV07-B2); genotype AB (subsequently described as AIV09) [5]; and genotype BB (subsequently described as AIV48). Whilst these genotypes are derived from the H5N1 detected during 2020/21 and into summer 2021 (Genotype C, AIV07-B1), where the AIV07-B2 genotype has maintained the same internal genes, the AIV09 is the result of a reassortment of the AIV07-B2 genotype with European AIVs present in wild birds gaining novel polymerase basic 2 (PB2) and polymerase acidic (PA) genes. In addition, the emergence of AIV48 in the UK in June 2022 was a further reassorted virus that acquired PA, nucleoprotein (NP) and non-structural (NS) genes from other wild bird strains including some known to be maintained in gulls (*Laridae*). However, the intra-genotype genetic diversity of the different genotypes is minimal regardless of geographical or temporal separation [5].

Avian influenza was presumed to be a major cause of the prolonged mass mortality of wild birds throughout the winter of 2021/22, on the Solway Firth. Significantly, this included substantial mortality in a species of conservation concern, as over winter, the Firth hosts the bulk of the global population of Svalbard Barnacle Geese (*Branta leucopsis*), where they exploit extensive estuarine and marine saltmarsh, mudflats and coastal fields (predominantly intensive pasture and short vegetation in arable land-uses). Waterbird species wintering on the Solway Firth benefit from multiple legal designations protecting the geographically extensive roosting and saltmarsh foraging sites around the Firth, on both the Scottish and English coasts (International designations: Special Protection Area; Ramsar. National designations: Site of Special Scientific Interest).

The geese breed and molt in Svalbard, then from late August begin their southward autumn migration along the west coast of Norway, arriving on the Solway Firth from mid-September to mid-October. Return passage usually begins in mid-April, with many pausing their northward migration in western Norway (Helgeland and Vesterålen) for several weeks, before onward flight back to Svalbard [6]. Due to protection from shooting and the establishment of reserves, the population of Barnacle Geese over-wintering on the Solway Firth has recovered from low numbers (~300 birds in 1948) to an estimated population of 39,700 geese for the 2020/2021 winter [7], although no mortality was associated with the 2020/2021 H5Nx UK outbreak. However, from early November 2021, Barnacle Geese, as well as many other species using the Firth, began to be found dead, with substantial numbers continuing to die over the successive months. The speed of the outbreak was so significant that there were concerns for the survival of the sub-population over-wintering on the Solway Firth. Within the Solway Firth region, H5N1 HPAI was also detected on six surrounding poultry premises, with these cases commonly linked to wild-bird incursions, and it was examined whether these poultry cases were linked to the mass die-off observed in Barnacle Geese. To better inform responses to these concerns, efforts were made to sample birds from across the area affected to understand the character of the outbreak (e.g., virus strain), predict the potential impact on the sub-population, and importantly identify evidence of any genetic specificity or evolution within the viral population which might indicate a specific threat to Barnacle Geese. This was possible given the nationwide description of concurrent circulating genotypes identified in other wild species across the UK [5].

## 2. Materials and Methods

Passive wild bird surveillance was undertaken primarily by the Department for the Environment, Food and Rural Affairs (Defra), utilizing collection services to deliver carcasses to regional APHA Veterinary Investigation Centres (VIC) via avian influenza helplines following reports of dead or sick birds from members of the public (https://www.gov.uk/guidance/report-dead-wild birds (accessed on 8 January 2024)). Birds were also submitted in collaboration with several conservation organizations (including the Wildfowl and Wetlands Trust (WWT) (Caerlaverock), NatureScot, and the Royal Society for the Protection of Birds (RSPB) Scotland). At the VIC, birds were swabbed (oropharyngeal (OP) and cloacal (C), where possible) and swabs were subsequently analysed at APHA Weybridge initially for the presence of AIV nucleic acid.

### 2.1. Virological Investigation

All swabs taken were placed individually in virus transport medium (either brain–heart infusion broth [8] or L-15 [9]) upon receipt in the laboratory before RNA was extracted and analysed for the presence of Influenza A viral nucleic acid using the matrix (M) gene specific real-time reverse-transcriptase PCR (rRT-PCR) [10]. Following identification of influenza A-positive samples, differential rRT-PCRs were used to subtype samples; here, the assays specific for the dominant circulating HA (H5) and NA (N1) genes [11,12] were used. Virus isolation was undertaken on selected H5N1 HPAIV positive clinical samples, where PCR positivity suggested potential for successful live virus recovery (Ct value ≤ 30), using 9- to 11-day-old, specified pathogen-free embryonated fowls’ eggs using standard methodologies [13].

### 2.2. Whole-Genome Sequencing and Phylogenetic Analysis

Following successful virus isolation, viral RNA was extracted and whole genome sequence (WGS) data were generated as previously described [5]. All genomes generated in this study have been uploaded to the GISAID EpiFlu Database (https://www.gisaid.org/ (accessed on 8 January 2024)) (Table 1). These sequences were combined with other contemporary global H5N1 haemagglutinin (HA) sequences and aligned using Mafft v7.487 [14] and were manually trimmed to define the open-reading frame and remove the signal peptide using AliView [15]. Phylogenetic trees were then inferred using the maximum-likelihood approach in IQ-Tree v2.1.4 [16] using ModelFinder [17] to infer the appropriate phylogenetic model and 1000 ultrafast bootstraps [18] and visualized as described previously [2,19]. Nucleotide comparisons were performed as described previously [19].

### 2.3. Serological Analysis

On 6 March 2023, 27 Svalbard Barnacle Geese were shot (under license 232327 for Science, Research and Education purposes granted by NatureScot) from a selected site along the Solway Firth for blood samples to be collected, gross post-mortem analysis and OP and C swabs taken. Sera were recovered from clotted blood collected and heat-treated at 56 °C for 30 min. Following heat-treatment, sera were analysed for the presence of H5-specific antibodies by Haemagglutination Inhibition (HI) assay using AIV H5N1 (A/Ck/Wales/053969/21), H5N3 (A/Teal/Eng/7394-2805/06) and H5N8 (A/Dk/Eng/036254/14) and as the test antigen [13]. HI titres with a reciprocal value equal to or greater than sixteen were taken to be positive.

## 3. Results

**Figure 1:**
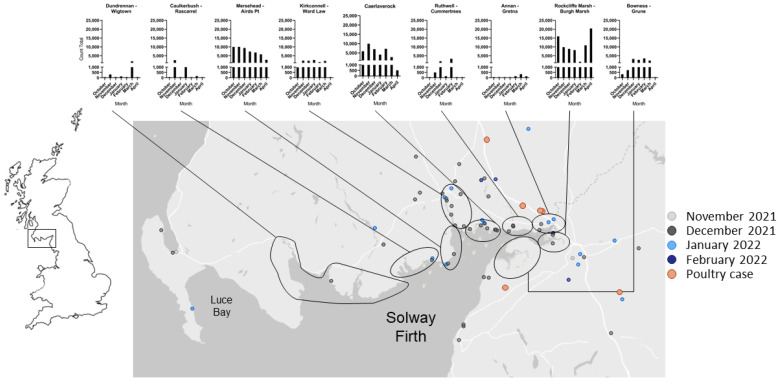
Location of wild birds testing positive for H5N1 HPAIV on the Solway Firth—Mapped position of H5 HPAI positive samples submitted to APHA for analysis. Regions of Barnacle Geese counts are shown with total counts for each region over the winter of 2021/2022 (adapted from [7]).

**Figure 2:**
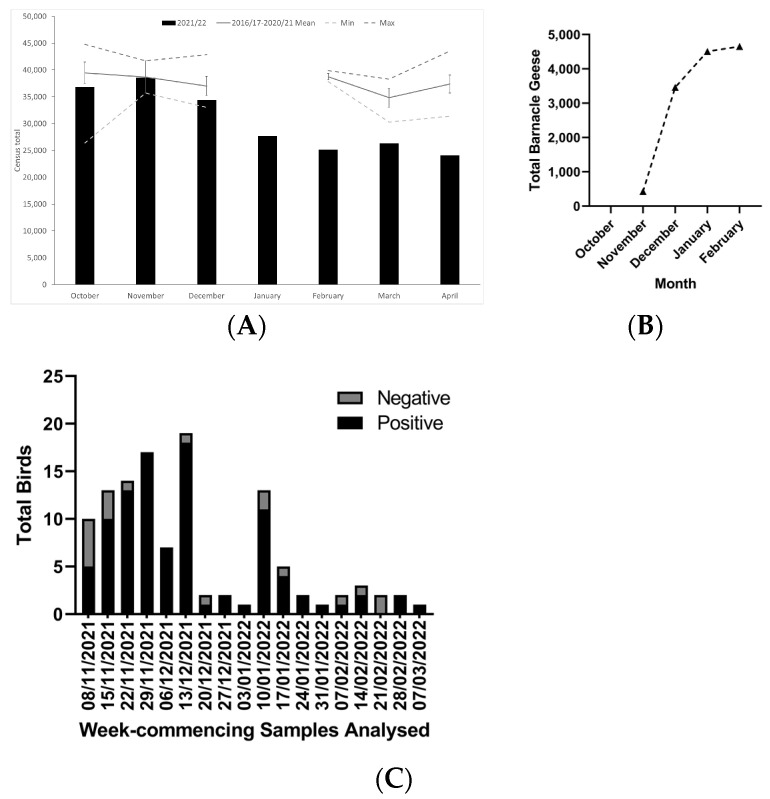
Barnacle Geese counts and samples submitted from Solway Firth region (**A**) total count of Barnacle geese from the Solway Firth region for 2022/23 and comparison to counts from previous years, (**B**) cumulative Barnacle Geese mortalities counted on the Solway Firth for 2021/2022, (**C**) positive and negative samples submitted to APHA for H5 HPAI analysis during winter 2021/2022 (A and B adapted from [7]).

Barnacle Geese returned to over-winter on the Solway Firth relatively late in 2021, with the first birds of Svalbard origin estimated to have arrived by approximately 10 October, with numbers then building rapidly to a Solway count of 33,183 birds on 13 October [7]. The peak count for the flyway population was noted the following month (36,185 birds on the Solway, count sites are shown in Figure 1), with an additional 2400 birds at Budle Bay, Northumberland (North-East England) on 10 November, consistent with numbers seen for previous years (Figure 2A). Numbers subsequently began to decline as dead birds were found and HPAI infection spread through the sub-population (Figure 2A). By 27 April (count 23,855 birds), the bulk of the population was at Rockcliffe Marsh on the southern, English side of the Solway (20,300 birds), with large numbers leaving the Solway by 4 May (count 13,200 birds); only a few birds remained by mid-May [20]. Using late season counts, it was estimated that total losses of Barnacle Geese due to HPAI were possibly as high as 13,200 birds, or 32% of the flyway population (Figure 2A) [7].

NatureScot collated voluntarily counts of carcasses along the Solway Firth from November 2021 including those from organizations such as RSPB, Natural England, WWT and ECO-LG Ltd. onwards. Carcasses were marked where possible to avoid double counting (spray paint), and a total of 4171 Barnacle Goose bodies were reported with a peak in deaths appearing to occur in December 2021 (Figure 2B), with a subsequent decline in mortality and no fresh carcasses reported in March or April, although this coincides with the departure of geese for their breeding grounds. Carcasses of other species were reported in much smaller numbers, with 49 Mute and Whooper Swans (*Cygnus olor* and *C. cygnus*) and 163 pink-footed geese (*Anser brachyrhynchus*), the next most numerous [7].

Following the initial detection of H5 HPAI in wild birds in Great Britain in late October 2021, the first HPAIV positive wild bird within the administrative areas surrounding the Solway Firth was a Barnacle Goose in Cumbria, testing positive for H5N1 HPAIV on 8 November 2021. Between 8 November 2021 and 7 March 2022, 116 birds from this study area (Figure 1) were submitted to APHA for AIV testing, with 86 birds of nine species testing positive for H5N1 HPAIV although not all birds appeared to succumb to H5 HPAIV infection (Table 2). Some birds (7 in total) could not be speciated (due to decomposition or predation). During November and early December, an increasing number of dead wild birds were submitted for AIV testing with a peak observed in the week commencing 13 December 2021, with a high proportion testing positive for H5 HPAI (Figure 2C). Following a lull in submissions, presumably due to the holiday period, a second peak was observed in January 2022 which contained the final Barnacle geese submitted from the Scottish-side of the Solway Firth (*n* = 2, both H5N1 HPAIV positive). Subsequently, numbers of submissions remained comparably low compared to November/December 2021. The final Barnacle Geese submitted from the English-side of the Solway Firth was in the week commencing 14 February 2022 (*n* = 2, both H5N1 HPAIV positive). A large proportion of birds submitted for AIV testing from the Solway Firth region were in coastal or estuarine locations, reflecting the distribution of Barnacle Geese and waterfowl in general across the study area (Figure 1). Birds found inland tended to be non-migratory resident species, e.g., Mute Swans or resident raptors such as Common Buzzards (Table 2). Barnacle Geese appeared to be the most susceptible species in this area, with 39 out of 41 Barnacle Geese (95%) testing positive for H5N1 HPAIV (Table 2), followed by Mute Swans (*n* = 17/20, 85% testing H5 HPAI positive) and Common Buzzards (*n* = 11/12, 92% testing H5 HPAI positive).

**Figure 3:**
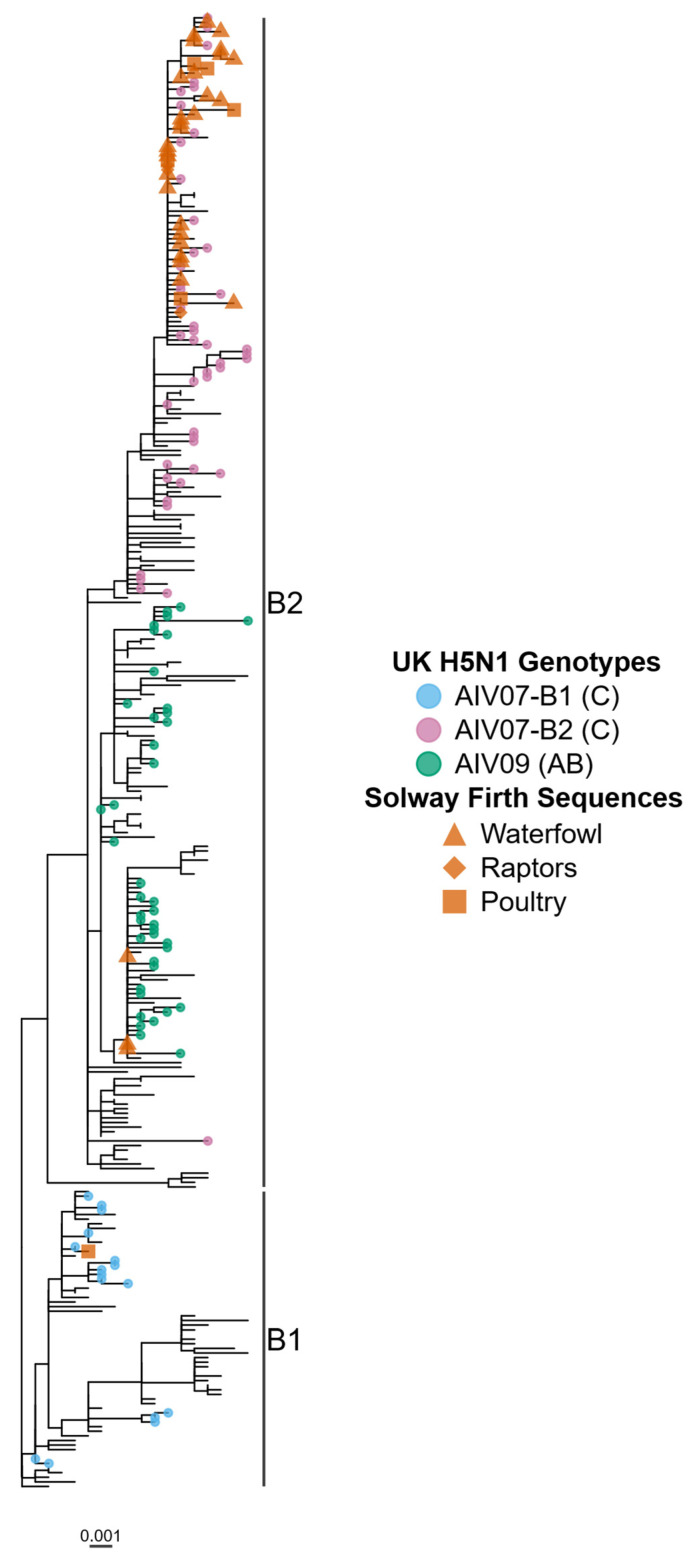
The HA of H5N1 HPAI isolated from birds on the Solway Firth are closely linked. A maximum-phylogenetic tree of the HA gene from H5N1 HPAI samples from United Kingdom between 2020 and 2022. The tips of UK samples are coloured according to genotype and Solway Firth bird type.

To further explore and understand the genetic composition of the AIVs detected in the Solway area, thirty-three samples from wild-bird submissions and a further six from outbreaks in poultry premises in the Solway Firth region were analysed by whole-genome sequencing. Samples sequenced were made-up from the following wild bird submissions, 30 were from waterfowl (24 Barnacle Geese, two Mute Swans, two Pink-footed Geese and two Whooper Swans), three were from raptors (two Common Buzzards and one Common Kestrel), and the remaining six sequences obtained from infected poultry (chicken) premises. Of the 33 wild birds, 30 were adult, two were immature, and the age of the final bird was indeterminate. Phylogenetic analysis of the haemagglutinin (HA) gene found that all 39 sequences belonged to the H5 clade 2.3.4.4b, with the cleavage site motif PLREKRRKRGLF, which is responsible for the ongoing global H5 HPAIV epizootic throughout 2021/2023. Further analysis of the HA sequences obtained from these samples found that 35 sequenced from the Solway Firth clustered within the H5N1 AIV07-B2 sub-lineage (Figure 3 and Figure S1) including all three sequences from raptors, 27 sequences from waterfowl and five sequences from poultry. The three sequences corresponding to the AIV-09 lineage were obtained from Barnacle Geese with samples collected between 12 and 17 November 2021, at the beginning of the mass die-off, with the remaining sequence obtained from a poultry premises in early December 2021, clustered within the B1 sub-lineage. These two H5 sub-lineages are both derived from the original H5N8 HPAIV incursion into Europe in late 2020 [21], but divergence was observed in the H5N1 HPAIV reassortant that came to be predominant from late 2021 [22]. Whilst in certain cases phylogenetic analysis found that a minority of sequences from the Solway Firth clustered together and demonstrated high sequence identity (HA sequences share greater than 98.79% nucleotide identity), for the most part, they show a high degree of similarity with other UK H5N1 sequences from 2021/22 with no geographical relationship (greater than 98.37% nucleotide identity for the HA). This further demonstrates the high degree of within-genotype similarity observed for the UK H5N1 sequences during the 2021/22 epizootic and suggests little to no genetic diversification had occurred within the Solway Firth mass die-off event, compared to sequences from the rest of the country, where some small levels of diversity had been observed [5].

Following the mass-mortality event on the Solway Firth in 2021/2022, we wished to examine the sera of returning Barnacle Geese in the winter 2022/2023 to examine whether H5 antibody responses had been induced as a result of exposure and infection with H5N1 HPAI, which would infer recovery from infection with an H5 virus presumed HPAI. Following the shooting of 27 protected Barnacle Geese by NatureScot (license 232327) from a selected site along the Solway Firth on 6 March 2023, blood samples were collected and analysed for the presence of anti-H5 antibodies. From 27 birds (male *n* = 14, female *n* = 13), blood samples were successfully collected from 25 birds, with 72% (*n* = 18) showing positive antibody response to H5 LPAI, suggesting recent infection (Table 3). However, when sera were measured against contemporary H5N1 HPAI, only 24% (*n* = 6) showed cross-reactivity, whilst 44% (*n* = 11) showed cross-reactivity against H5N8 AIV. Swabs taken recovered no positive signals for viral RNA, whilst no clear signs of infection commonly observed following H5N1 HPAI infection were observed following post-mortem examination [23]. It should be noted that in the 2022/2023 HPAI outbreak in GB, only two Barnacle Geese tested positive for H5N1 HPAI from the Solway Firth region, suggesting that those returning had a partial amnestic response to the circulating virus. Experimental analysis of wild ducks demonstrated that prior exposure to H5 HPAIV was sufficient to prevent shedding of live H5 HPAIV a year later [24]. In contrast to the Greenland Barnacle Geese that over-winter predominantly on the Isles of Argyll and Bute and Ireland, in areas to the west and northwest of the Solway (c.100 km minimum between wintering ranges), where increased mortality was observed in the winter of 2022/23 (November–February) in comparison to 2021/2022.

## 4. Discussion

The mass mortality of Barnacle Geese on the Solway Firth from H5N1 HPAI resulted in an estimated mortality rate of ~30%, whilst other migratory species (e.g., Pink-footed Geese) and native species including Mute Swans and Common Buzzards also saw large mortality rates in this region. Mass mortalities of species not normally known to harbour H5N1 infections have also been observed in other species throughout the UK and Europe [25,26,27]. This mass mortality event on the Solway Firth has given a unique opportunity to examine both the kinetic profile of deaths throughout such an event and the genetic variation of the virus within this population, in part because the geese represent a defined sub-population of conservation concern and had thus been closely monitored for years before the HPAIV epizootic. Knowledge of the abundance and distribution of the birds allowed workers to quickly identify the scale and severity of goose mortality, and informed carcass counts, and sampling activities should be focused on.

In principle, the abundance of Barnacle Geese on the Solway Firth, and their habit of aggregating at significant density in foraging habitats suggested that the introduction of a highly virulent and contagious virus such as HPAIV might be followed by rapid establishment, spread and mortality across the population. It is intriguing therefore that this closely monitored population did not show evidence of any significant mortality throughout the previous epizootic season, with no observed detections of H5 HPAI within the Solway Firth region during 2020/2021, suggesting a large, mostly naïve population of birds. Due to the devastating nature of the 2021/22 H5N1 strain of HPAI, this resulted in a mass die-off of Barnacle Geese, along with increased mortalities in other waterfowl species and raptors within the Solway Firth region, with dead birds submitted on multiple occasions for testing. Official counting of Barnacle Goose carcasses is, however, likely to have resulted in a substantial underestimation of the true mortality, as coverage was concentrated in specific areas, but was largely ad hoc in others, with some large areas of coastal marsh remaining too remote for safe access. Other reasons included the tidal effect of the estuary on the number of carcasses found; the wetlands and saltmarshes of the Solway Firth being difficult habitats to search; and birds that died inland were not necessarily counted and some groups, e.g., farmers had no organized reporting mechanism and sometimes reported burying carcasses. Evidence of the tidal removal of carcasses from the Solway included not only small numbers of ad hoc counts of dead birds received from sites along the west coast of Cumbria but also the signals received from three GPS collars on dead birds (two Barnacle Geese and one Pink-footed Goose). Noting these caveats, the sites with most of the reported number of carcasses were within reserves, the Solway Barnacle Goose Management Scheme Area and the coastal strips near the main roost sites, e.g., around Mersehead, West Preston, Southerness, Caerlaverock, Priestside and Rockcliffe Marsh (Figure 1). However, the Solway Barnacle Geese were not the only mass die-off event observed in wild birds within the UK during this outbreak with coastal sea birds, specifically Northern Gannets (*Morus bassanus*) experiencing significant die-off events [1]. It is therefore clear that this strain of HPAIV is highly transmissible within and between wild bird populations, causing devastating effects within established population groups.

Across the UK, the genetic diversity of H5N1 HPAIV has been mapped, with the H5 originally deriving from an H5N8 virus detected in May 2020 [21], but with further diversification into two HA sub-lineages observed during the 2022 season throughout Europe [18]. Although there have been multiple genotypes described, the two most frequently detected throughout the UK have been designated AIV07-B2 (Genotype C) and AIV09 (Genotype AB) [5]. Analysis determined that the initial detected incursion of H5N1 HPAIV into the Solway Firth in early November 2021 was of the AIV07-B2 genotype of the virus (A/barnacle_goose/England/292152021; 8 November). The AIV07-B2 genotype was detected in the majority of sequences generated from this selection of samples; however, the H5N1 AIV09 genotype was also detected from the 12 November onwards (A/barnacle_goose/Scotland/292583/2021), but only in a minority of the samples from the Solway Firth (*n* = 3). This may suggest either that the AIV07-B2 genotype has a replicative advantage in waterfowl and particularly Barnacle Geese, or this genotype was the initial isolate introduced into this population (*n* = 11) in late November and was therefore able to spread quickly amongst the Barnacle Geese population. Analysis of wild bird genomes from other regions of the UK suggest an approximately equitable division between the AIV07-B2 and AIV09 genotypes, although the relative fitness for AIV07-B2 and AIV09 genotypes in geese would need to be assessed. Within this group of sequences, the genetic diversity of the AIV07-B2 isolates were also examined, and it appeared that across the eight gene segments, there was a high degree of genetic identity (greater than 99.4% nucleotide identity), suggesting there was no evidence of adaptation within specific hosts as the virus spread through the population. Adaptation may not be necessary due to the large number of naïve hosts, but it may be in later years, where surviving birds are able to respond to the virus better, and host-specific adaptation may subsequently be observed. It may therefore be prudent to follow virus isolates recovered for Barnacle Geese from the Solway Firth in the subsequent years due to the short time frame (2 months) that the virus was examined in this study.

The UK and Europe poultry industry suffered considerably from the H5N1 HPAI outbreak. In the 2022 epizootic event, there were 119 incursions of H5N1 HPAI on commercial poultry premises (to 8 July 2022; Animal and Plant Health Agency, 2022), of which six were within our wider study area (Dumfries and Galloway in Scotland and Cumbria in England). Five of these produced viral sequences that were closely related to those concurrent in the wild bird population (AIV07-B2), supporting the association between disease in wild birds and commercial poultry HPAI (see Figure 3). This again highlights the necessity of good biosecurity practices on commercial premises to mitigate against incursion.

A further problem observed during this mass mortality event is the presence of a high concentration of infected carcasses, leading to the potential environmental contamination of sites. It is therefore difficult to distinguish whether the mass mortality event observed was due to direct bird-to-bird transmission or whether it was due to the environmental contamination of the large numbers of dead carcasses remaining on the site. A further issue that arose from the large numbers of carcasses of dead birds was the potential onward infection to raptors. During the 2021–2022 H5N1 epizootic, raptors, including Common Buzzard (*Buteo buteo*), Common Kestrel (*Falco tinnunculus*), Peregrine Falcon (*Falco peregrinus*) and White-tailed Sea Eagle (*Haliaeetus albicilla*) all tested positive for the detection of H5N1 HPAI vRNA [4], which would be hypothesized to be transmitted through the ingestion of contaminated carcasses. Along the Solway Firth, eleven Common Buzzards and a single Common Kestrel were infected with H5N1 HPAI, with isolated virus being phylogenetically linked to those observed in waterfowl (Figure 3). It is therefore highly likely that the onward transmission observed in raptors in this area was due to the consumption of contaminated carcasses.

Although the Svalbard Barnacle Goose population has been stable for the past few years, it is still classified as Amber on the UK Birds of Conservation Concern list due to non-breeding localization [28]. The substantial mortality observed with the Svalbard Barnacle Goose population reported here is anticipated to have had a significant impact on this population, with the full impact potentially not known for many years. It is of interest to note that a second well-monitored separate sub-population of Barnacle Geese from a non-overlapping flyway, birds that breed in Iceland and Greenland and over-winter principally on Islay (western Scotland) and the west coast of Ireland were affected to a far lesser extent, and later, with only limited mortality observed over the 2022 winter in February and March 2022. The reasons for this are not clear; however, there is the potential that in 2021 the Svalbard Barnacle Geese arrived at an area which already had a virus within the region. Indeed, the initial cohort of birds submitted for AI testing from Cumbria and Dumfries and Galloway were mostly swans (Mute and Whooper), with only four of the initial 16 birds testing positive for H5N1 HPAI being Barnacle Geese. It therefore appears likely that the virus was already present when this population arrived. Interestingly, during the 2020/21 H5N8 epizootic, there were no recorded deaths on the Solway Firth due to HPAI. Epidemiological analysis appears to show that the current H5N1 HPAI is able to infect a broader range of species [1,29], resulting in prolonged transmission within the wild bird populations and increased environmental contamination, ultimately resulting in increased infectivity. In the subsequent 2022/23 winter, however, the mortality of Svalbard Barnacle Geese was very low, but in the Greenland Barnacle Geese it escalated markedly [4], raising further questions about the roles of immunity, interplay of viral variants and environmental persistence. This low level of observed mortality on the Solway Firth during the 2022/23 winter may be due to the observed positive antibody response to H5 LPAIV in a small sample size of Barnacle Geese shot, with 72% showing a positive response, although only 24% of the geese were positive against H5N1 HPAI antigen, with a further 16% (*n* = 4) birds showing responses below the positive cut-off, but this may be that a high percentage of birds need to have high levels of antibodies against H5N1 HPAI for viral spread to be significantly reduced. Ideally, it would therefore be of interest to follow antibody responses of Barnacle Geese returning to the Solway Firth annually, ideally through capture, sampling and release methodologies, and where possible simultaneously with ringing of birds to allow for the identification of birds in each season. The kinetics of antibody responses in wild birds are poorly understood, although analysis of the Northern Gannet (*Morus bassanus*) population, which was also heavily affected H5N1 HPAIV during the spring/summer (May–July) of 2022 did show H5-antibody-positive birds [25]. It would also be useful to analyse an increased number of differing bird species from this region to determine the resultant response to the 2022 outbreak, and how many of the surviving birds were able to mount an antibody response to the currently circulating virus to provide insights to infection dynamics within a single ecosystem. A further question arising from the analysis of sera is the birds’ ages. We noted that the vast majority of birds where H5N1 HPAIV RNA was detected were adults (adult = 89% (*n* = 87/98), immature = 7% (*n* = 7/98), and unknown age = 4% (*n* = 4/98)), although it is difficult to determine the age of carcasses submitted; however, the average lifespan of a Barnacle Goose is thought to be 14 years, with some reaching almost 27 years of age [30]. It therefore suggests that these birds did not have immunity prior to the 2022 outbreak, and this may have been the first time that the birds were infected with H5 HPAIV. This is backed up by the fact that, as stated earlier, there were no reported deaths in the Solway Firth region during 2020/2021 outbreak. It would therefore, where possible, be useful in future analysis to try to determine the ages of the bird carcasses to ascertain the role of age on susceptibility to incoming H5 HPAIV. It does, however, appear that the serological responses examined in the winter of 2022/23 give a strong indication that those birds surviving the mass mortality event are now able to mount a strong anamnestic response to H5N1 HPAI and reduce the burden on young, immature birds. This is borne-out by the fact that, as stated previously, H5N1 HPAIV was detected in only two Barnacle Geese from the Solway Firth during the 2022/23 winter.

Overall, the genetic analysis demonstrates that this H5N1 HPAIV is a genetically stable virus within the host Barnacle Goose population, with limited mutations and with no apparent reassortment events observed from the samples studied, but the analysis does suggest at least two independent introductions into this region.

## Figures and Tables

**Table 1:** Swab samples selected for whole-genome sequencing analysis.

Town	Country	Date Collected	Species	Sample ID	GISAID Ref	Age
Carlisle	England	8 November 2021	Barnacle Goose	A/barnacle_goose/England/292151/2021	EPI_ISL_13369744	Immature
Caerlaverock	Scotland	9 November 2021	Whooper Swan	A/Whooper_swan/Scotland/056219/2021	EPI_ISL_9029962	Unknown
Caerlaverock	Scotland	12 November 2021	Barnacle Goose	A/barnacle_goose/Scotland/292583/2021	EPI_ISL_13369753	Adult
Annan	Scotland	16 November 2021	Barnacle Goose	A/Barnacle_goose/Scotland/058494/2021	EPI_ISL_13453561	Adult
Caerlaverock	Scotland	16 November 2021	Common Kestrel	A/Kestrel/Scotland/058512/2021	EPI_ISL_13369761	Immature
Castle Douglas	Scotland	16 November 2021	Pink-footed goose	A/pink-footed_goose/Scotland/058488/2021	EPI_ISL_13369760	Adult
Caerlaverock	Scotland	17 November 2021	Barnacle Goose	A/barnacle_goose/England/061307/2021	EPI_ISL_13369762	Adult
Ruthwell	Scotland	19 November 2021	Barnacle Goose	A/barnacle_goose/Scotland/062003/2021	EPI_ISL_13369765	Adult
Annan	Scotland	19 November 2021	Barnacle Goose	A/barnacle_goose/Scotland/062007/2021	EPI_ISL_13369766	Adult
Silloth	England	26 November 2021	Barnacle Goose	A/barnacle_goose/England/293824/2021	EPI_ISL_13369779	Adult
Carlisle	England	26 November 2021	Barnacle Goose	A/barnacle_goose/England/293809/2021	EPI_ISL_13369780	Adult
Kirkcudbright	Scotland	30 November 2021	Barnacle Goose	A/barnacle_goose/Scotland/064368/2021	EPI_ISL_13370417	Adult
Kirkcolm	Scotland	1 December 2021	Common Buzzard	A/common_buzzard/Scotland/294149/2021	EPI_ISL_13370418	Adult
Allerdale	England	1 December 2021	Mute Swan	A/Mute_Swan/England/294146/2021	EPI_ISL_13370506	Adult
Silloth	England	4 December 2021	Barnacle Goose	A/barnacle_goose/England/294327/2021	EPI_ISL_13370517	Adult
Annan	Scotland	4 December 2021	Barnacle Goose	A/Barnacle_goose/Scotland/075760/2021	EPI_ISL_13486748	Adult
Caerlaverock	Scotland	4 December 2021	Barnacle Goose	A/Barnacle_goose/Scotland/075827/2021	EPI_ISL_13486760	Adult
Annan	Scotland	4 December 2021	Mute Swan	A/Mute_swan/Scotland/075821/2021	EPI_ISL_13486881	Adult
Annan	Scotland	6 December 2021	Barnacle Goose	A/barnacle_goose/Scotland/072143/2021	EPI_ISL_13370522	Adult
Allerdale	England	9 December 2021	Barnacle Goose	A/barnacle_goose/England/294620/2021	EPI_ISL_13370561	Adult
Caerlaverock	Scotland	10 December 2021	Barnacle Goose	A/Barnacle_goose/Scotland/075563/2021	EPI_ISL_13486738	Adult
Caerlaverock	Scotland	11 December 2021	Barnacle Goose	A/Barnacle_goose/Scotland/072168/2021	EPI_ISL_13486729	Adult
Caerlaverock	Scotland	11 December 2021	Whooper Swan	A/Whooper_swan/Scotland/072171/2021	EPI_ISL_13486975	Adult
Dumfries	Scotland	13 December 2021	Barnacle Goose	A/Barnacle_goose/Scotland/072140/2021	EPI_ISL_13453562	Adult
Annan	Scotland	13 December 2021	Barnacle Goose	A/Barnacle_goose/Scotland/072152/2021	EPI_ISL_13453563	Adult
Thornhill	Scotland	15 December 2021	Common Buzzard	A/Common_buzzard/Scotland/073099/2021	EPI_ISL_13486819	Adult
Dumfries	Scotland	17 December 2021	Barnacle Goose	A/Barnacle_goose/Scotland/109090/2021	EPI_ISL_13486785	Adult
Dumfries	Scotland	17 December 2021	Barnacle Goose	A/Barnacle_goose/Scotland/109105/2021	EPI_ISL_13486796	Adult
Ruthwell	Scotland	27 December 2021	Barnacle Goose	A/Barnacle_goose/Scotland/003169/2021	EPI_ISL_13486688	Adult
Dumfries	Scotland	30 December 2021	Barnacle Goose	A/Barnacle_goose/Scotland/076711/2021	EPI_ISL_13486772	Adult
Clarencefield	Scotland	31 December 2021	Barnacle Goose	A/Barnacle_goose/Scotland/003588/2022	EPI_ISL_13486713	Adult
Port Logan	Scotland	2 January 2022	Barnacle Goose	A/Barnacle_goose/Scotland/003579/2022	EPI_ISL_13486700	Adult
Troqueer	Scotland	8 January 2022	Pink-footed goose	A/pink-footed_goose/Scotland/003617/2022	EPI_ISL_13782461	Adult

**Table 2:** HPAIV positive species and numbers submitted for AI testing to APHA from the Solway region.

Species	Number Tested	Positive	% Positivity	Number of Samples Submitted for WGS
Barnacle Goose (*Branta leucopsis*)	43	41	95.35	24
Mute Swan (*Cygnus olor*)	20	17	85	2
Common Buzzard (*Buteo buteo*)	12	11	91.67	2
Whooper Swan (*Cygnus cygnus*)	7	7	100	2
Pink-footed Goose (*Anser brachyrhynchus*)	6	5	83.33	2
Greylag Goose (*Anser anser*)	5	5	100	
*Pheasant* (*Phasianus colchicus*)	3	2	66.67	
Common Starling (*Sturnus vulgaris*)	3	0	0	
Canada goose (*Branta canadensis*)	2	2	100	
Indian runner duck (*Anas platyrhynchos domesticus*)	2	0	0	
Kestrel (*Falco tinnunculus*)	2	1	50	1
Hen Harrier (*Circus cyaneus*)	1	0	0	
Herring Gull (*Larus argentatus*)	1	0	0	
Eurasian Sparrowhawk (*Accipiter nisus*)	1	0	0	
Tawny Owl (*Strix aluco*)	1	0	0	
Goose unspecified	5	5	100	
Gull unspecified	1	1	100	
Swan unspecified	1	1	100	
Grand Total	116	98		33

**Table 3:** Serological response by barnacle geese from the Solway Firth shot 6 March 2023 including HI titre, result of HI analysis and sex of the Barnacle Goose.

Sample Number	H5N1		H5N8		H5N3		Sex
	Titre	Result	Titre	Result	Titre	Result	
1	<2	Negative	8	Negative	128	Positive	F
2	8	Negative	16	Positive	32	Positive	F
3	16	Positive	32	Positive	64	Positive	F
4	128	Positive	64	Positive	256	Positive	M
5	<2	Negative	2	Negative	32	Positive	F
6	2	Negative	8	Negative	512	Positive	F
7	2	Negative	8	Negative	64	Positive	F
8	2	Negative	32	Positive	256	Positive	F
9	16	Positive	8	Negative	4	Negative	M
10	8	Negative	8	Negative	512	Positive	M
11	128	Positive	32	Positive	512	Positive	F
12	<2	Negative	16	Positive	32	Positive	F
13	IS	ND	IS	ND	IS	ND	M
14	IS	ND	IS	ND	IS	ND	M
15	<2	Negative	<2	Negative	<2	Negative	M
16	<2	Negative	<2	Negative	256	Positive	M
17	<2	Negative	32	Positive	256	Positive	F
18	4	Negative	32	Positive	64	Positive	M
19	2	Negative	8	Negative	8	Negative	F
20	<2	Negative	<2	Negative	32	Positive	F
21	<2	Negative	<2	Negative	<2	Negative	M
22	<2	Negative	<2	Negative	<2	Negative	M
23	8	Negative	32	Positive	128	Positive	M
24	<2	Negative	8	Negative	<2	Negative	F
25	64	Positive	64	Positive	128	Positive	M
26	<2	Negative	<2	Negative	<2	Negative	M
27	32	Positive	16	Positive	64	Positive	M

IS: Insufficient sera. ND: Not defined.

## Data Availability

The sequences generated from the current study were deposited in the Global Initiative on Sharing All Influenza Data (GISAID) EpiFlu™ database.

## Supplementary Materials

The following supporting information can be downloaded at https://www.mdpi.com/article/10.3390/pathogens13010083/s1. **Figure S1**: Phylogenetic analysis of HA from H5N1 HPAI isolated from birds on the Solway Firth.
