# Supplementary figures and images for: Genetic Analysis of H5N1 High-Pathogenicity Avian Influenza Virus following a Mass Mortality Event in Wild Geese on the Solway Firth
Source: Pathogens. 2024 Jan 17;13(1):83. doi: 10.3390/pathogens13010083 (PMC10818813; doi:10.3390/pathogens13010083)

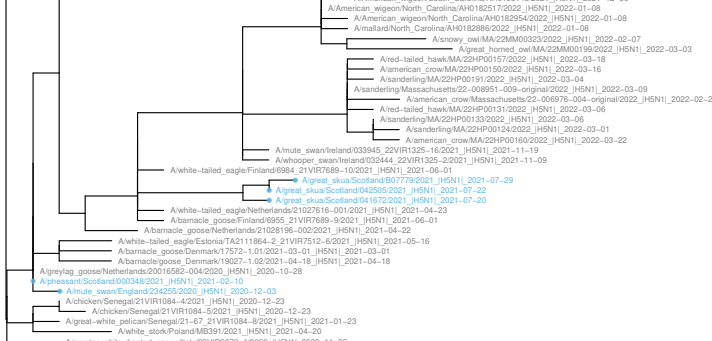

B2

## UK H5N1 Genotypes

- AIV07-B1 (C)
- AIV07-B2 (C)
- AIV09 (AB)

# Solway Firth Sequences

- ▲ Waterfowl
- ◆ Raptors
- Poultry

B1

Supplement: Supplementary file 1 [file pathogens-13-00083-s001.zip › pathogens-2768997-supplementary.pdf]
